# Successful Low-Dose Oxytocin Induction of Labor at 40+5 Weeks in a Primigravida With Prior Laparoscopic Myomectomy Preserving the Endometrial Cavity: A Case Report

**DOI:** 10.7759/cureus.113517

**Published:** 2026-07-28

**Authors:** Niko Koridze, Saba Barbakadze, Mariam Morchadze, Alexandra Barnovi, Giorgi Patsatsia

**Affiliations:** 1 Obstetrics and Gynaecology, Zhordania Medical Center, Tbilisi, GEO; 2 Obstetrics and Gynaecology, David Tvildiani Medical University, Tbilisi, GEO; 3 Pediatrics, University of Georgia, Tbilisi, GEO; 4 Obstetrics and Gynaecology, Tbilisi State Medical University, Tbilisi, GEO

**Keywords:** fibroid, induction of labor, labor induction, laparoscopic myomectomy, myomectomy, oxytocin, primigravida, uterine rupture, uterine scar

## Abstract

Induction of labor in women with a prior uterine scar from myomectomy remains a clinically challenging decision, primarily due to concerns about uterine rupture. The risk of rupture varies substantially depending on whether the endometrial cavity was entered during surgery, the depth of myometrial involvement, and the suturing technique employed. We report a case of a 30-year-old primigravida (G1P0) who had undergone laparoscopic myomectomy three years prior for a 5×3 cm subserosal-intramural fibroid, with multilayer uterine closure and without breach of the endometrial cavity. Following membrane sweeping at 39 weeks of gestation, spontaneous labor did not ensue. At 40+5 weeks, with a Bishop score exceeding six and cervical dilatation of 4 cm, oxytocin induction was commenced using a low-dose protocol (30 units in 500 mL). The patient delivered vaginally within 10 hours of oxytocin initiation. The neonate was born in good condition, weighing 3,130 g, measuring 51 cm, with APGAR (Appearance, Pulse, Grimace, Activity, and Respiration) scores of 9/10 and umbilical arterial cord pH of 7.32. No uterine rupture, neonatal morbidity, or other significant maternal complications were observed. This case supports the feasibility of low-dose oxytocin induction in carefully selected women with a prior laparoscopic myomectomy when the endometrial cavity was not entered and multilayer closure was performed, and contributes to the growing body of evidence that individualized intrapartum management may be appropriate in this setting.

## Introduction

Uterine leiomyomas are the most common benign tumors of the female reproductive tract, affecting up to 70% of women of reproductive age [1]. In women who wish to preserve fertility, myomectomy - whether by laparotomy or laparoscopy - remains the standard surgical approach [2]. Laparoscopic myomectomy (LM) has become increasingly preferred over abdominal myomectomy due to its advantages of reduced blood loss, faster recovery, and lower rates of postoperative adhesion formation, all of which may benefit subsequent reproductive outcomes [2].

However, LM creates a uterine scar, and subsequent pregnancy raises concerns about scar integrity during labor, particularly the risk of uterine rupture - a rare but potentially catastrophic complication. Systematic reviews have estimated the overall risk of uterine rupture following myomectomy at approximately 1%, a figure comparable to that reported for trial of labor after cesarean delivery [3,4]. Several factors have been implicated in myometrial healing and subsequent rupture risk, most notably whether the endometrial cavity was breached during surgery, the depth of myometrial involvement, and the adequacy of uterine closure technique [3,5]. Additional factors thought to influence the healing process include the use of electrosurgical energy during fibroid excision and its effect on local tissue vascularization, the size and number of fibroids removed, the interval between surgery and subsequent conception, and postoperative complications such as hematoma or infection. In the present case, several of these favorable conditions were met: the endometrial cavity remained intact, a multilayer closure technique was employed, and a sufficient interval had elapsed between myomectomy and conception, all of which informed our decision to permit a trial of labor.

The question of how to manage labor and delivery after LM is therefore a matter of ongoing clinical debate. Survey data indicate that the majority of obstetricians are willing to permit a trial of vaginal labor when the endometrial cavity was not entered during myomectomy (approximately 71%-76%), compared with far fewer (14%-27%) when cavity entry did occur [5]. The American College of Obstetricians and Gynecologists (ACOG) recommends elective cesarean section when the endometrial cavity was entered, but does not categorically preclude vaginal delivery in all post-myomectomy patients [3]. The additional question of whether oxytocin induction is safe in women with a post-myomectomy uterine scar remains poorly studied, with most available evidence extrapolated from the trial of labor after cesarean literature, in which high-dose oxytocin has been associated with increased rupture risk [6,7].

We present a case of successful low-dose oxytocin induction of labor in a primigravida at 40+5 weeks' gestation with a prior laparoscopic myomectomy in which the endometrial cavity was preserved and multilayer uterine closure was performed. This case illustrates the clinical decision-making process and intrapartum course in this uncommon but increasingly encountered clinical scenario.

## Case presentation

A 30-year-old primigravida (G1P0) presented at 40+5 weeks of gestation for obstetric assessment. Her past gynecological history was significant for a laparoscopic myomectomy performed three years prior for a symptomatic FIGO (International Federation of Gynecology and Obstetrics staging system) type 6 (intramural with subserosal extension) uterine fibroid measuring 5×3 cm, presenting clinically with pelvic pain and dysmenorrhea attributable to myometrial distortion from the intramural component. The operative record confirmed that the fibroid excision did not extend into the endometrial cavity, consistent with its FIGO type 6 classification. Uterine reconstruction was performed using interrupted sutures in a multilayer closure technique; the suture material, gauge, and exact number of layers were not specified in the available operative documentation. The postoperative course was uncomplicated, and no intraoperative or early postoperative complications were documented.

The current pregnancy was uncomplicated. Routine antenatal surveillance included ultrasound assessment of the myomectomy scar, which was located on the anterior uterine wall and therefore well visualized throughout pregnancy; no abnormality, including thinning or dehiscence, was identified at any point during the antenatal period. At 39+0 weeks, membrane sweeping was performed in an attempt to promote spontaneous labor. However, no spontaneous uterine contractions or cervical change sufficient to initiate labor occurred in the subsequent two weeks.

At 40+5 weeks, the patient was reassessed. Cervical examination revealed a Bishop score exceeding 6, with cervical dilatation of 4 cm, consistent with a favorable cervix. Following detailed counseling regarding the risks and benefits of induction of labor in the setting of a prior uterine scar, including the risk of uterine rupture, the patient provided informed consent for oxytocin induction.

Induction was initiated with a low-dose oxytocin infusion (30 units in 500 mL normal saline, 60 mU/mL), commenced at a starting rate of 1 mL/hour (1 mU/min) and titrated with incremental increases of 1 mL/hour (1 mU/min) every 30 minutes according to uterine response. This regimen was consistent with the ACOG-defined low-dose oxytocin protocol (starting dose 0.5-2 mU/min, increments of 1-2 mU/min every 15-40 minutes). Continuous cardiotocographic (CTG) monitoring was maintained throughout the induction and active labor period. No pathological CTG changes, uterine tachysystole, or clinical signs suggestive of uterine scar compromise were observed at any point during the induction or labor.

The patient achieved active labor and progressed to a spontaneous vaginal delivery 10 hours after the commencement of the oxytocin infusion. The third stage of labor was uncomplicated. Manual examination of the uterine cavity was performed following placental delivery and confirmed uterine integrity, with no evidence of scar dehiscence or rupture.

A female neonate was delivered in good condition, with a birth weight of 3,130 g and a length of 51 cm. The APGAR (Appearance, Pulse, Grimace, Activity, and Respiration) scores were nine at one minute and 10 at five minutes. Umbilical arterial cord blood pH was 7.32, within the normal range. No neonatal resuscitation was required, and no neonatal morbidity was identified. Both mother and neonate were discharged in satisfactory condition.

## Discussion

This case illustrates a successful outcome following low-dose oxytocin induction of labor in a primigravida with a prior laparoscopic myomectomy, where the endometrial cavity was preserved intact and multilayer uterine closure was employed. Several key clinical features of this case merit specific discussion.

Risk stratification and the role of endometrial cavity integrity

The single most important factor cited in the literature for determining appropriate mode of delivery and labor management after myomectomy is whether the endometrial cavity was entered during surgery [3,5]. Entry into the cavity is associated with a greater depth of myometrial disruption, potentially compromising scar integrity and increasing the risk of rupture during labor. Survey data from obstetricians confirm this: willingness to allow vaginal delivery drops from approximately 71%-76% when the cavity is preserved to only 14%-27% when it was breached, regardless of whether the surgical approach was laparoscopic or open [5]. In the present case, the operative record explicitly documented that the fibroid - a FIGO type 6 lesion measuring 5×3 cm - was excised without entry into the endometrial cavity, placing this patient in the lower-risk category for scar-related complications.

Multilayer uterine closure and surgical interval

Multilayer closure of the hysterotomy defect following myomectomy is considered best practice and has been associated with improved scar healing compared with single-layer repair [2,3]. In the present case, the documented multilayer suturing technique provides further reassurance regarding scar integrity. Additionally, the interval between the myomectomy and the index pregnancy was three years, well beyond the generally recommended minimum interval of six to 12 months before conception following myomectomy [2]. An adequate interval between surgery and pregnancy allows sufficient time for complete myometrial scar healing and remodeling, which may further reduce the risk of rupture [2,8].

Low-dose oxytocin and uterine scar safety

The use of oxytocin in women with a scarred uterus has attracted significant attention in the context of trial of labor after cesarean section (TOLAC). Meta-analyses of TOLAC populations have reported higher rates of uterine rupture with induced labor compared with spontaneous labor, and oxytocin has been implicated as a contributing factor, particularly at high doses or when used in the latent phase [6,7]. Evidence from case reports and case series has also highlighted that uterine rupture following myomectomy has in some instances occurred in the context of high-dose oxytocin administration [3]. However, the evidence base specific to oxytocin use after myomectomy (as distinct from cesarean scar) is very limited.

In the present case, a low-dose protocol was deliberately chosen: oxytocin was administered as 30 units in 500 mL normal saline (60 mU/mL), commenced at a starting rate of 1 mL/hour (1 mU/min) and titrated with increments of 1 mL/hour (1 mU/min) every 30 minutes. This regimen is consistent with the ACOG-defined low-dose oxytocin protocol (starting dose 0.5-2 mU/min, increments of 1-2 mU/min every 15-40 minutes), and we detail it explicitly here to allow readers to contextualize the protocol used against established low-dose definitions and to support reproducibility in similar cases. The starting point of an already favorable cervix (Bishop score >6, dilatation 4 cm) meant that only modest augmentation was required to achieve active labor. The 10-hour labor-to-delivery interval, normal CTG throughout, and absence of uterine hyperstimulation suggest that the myometrium responded appropriately to the oxytocin stimulus without undue stress on the scar. The favorable cervical status at the time of induction was itself an important permissive factor: an unfavorable cervix requiring prolonged or high-dose oxytocin exposure to achieve cervical ripening would represent a substantially higher-risk scenario [7].

Membrane sweeping as a preparatory intervention

Membrane sweeping was performed at 39+0 weeks in this patient. Although this did not initiate spontaneous labor in the two-week interval before induction, it may have contributed to the cervical ripening that was evident at 40+5 weeks. Membrane sweeping is a well-established mechanical method for promoting cervical change and reducing the need for formal induction of labor; its use in women with a uterine scar is generally not contraindicated and carries no specific rupture risk [8].

Intrapartum monitoring and uterine integrity

Continuous CTG monitoring was maintained throughout labor in accordance with recommended practice for women with a uterine scar. The absence of any pathological fetal heart rate changes, particularly late decelerations or a sinusoidal pattern, provided reassurance that uteroplacental function was maintained and that no acute scar compromise occurred. Post-delivery manual uterine exploration confirmed structural uterine integrity, providing objective confirmation of the absence of scar dehiscence or rupture.

Neonatal outcome

The neonate was delivered in excellent condition, with a birth weight of 3,130 g and APGAR scores of nine and 10 at one and five minutes, respectively. An umbilical arterial cord pH of 7.32 indicates no intrapartum acidemia, reflecting well-preserved fetal oxygenation throughout labor. These outcomes are reassuring and consistent with the uncomplicated intrapartum course.

Clinical implications

This case supports the view that, in carefully selected patients - specifically those with a prior laparoscopic myomectomy where the endometrial cavity was preserved, multilayer closure was performed, an adequate surgical interval has elapsed, and the cervix is favorable at the time of induction - low-dose oxytocin induction of labor may represent a safe and viable option. Delivery in this case took place at Zhordania Medical Center, a secondary care center with tertiary-level obstetric capabilities, including round-the-clock availability of emergency cesarean delivery and neonatal support. We recommend that women with a prior myomectomy scar who are candidates for induction of labor be managed at a center with equivalent capabilities, given that prompt access to emergency cesarean delivery is an essential safety requirement in this setting. Individualized risk assessment, thorough informed consent, continuous intrapartum CTG monitoring, and delivery at a facility capable of immediate emergency cesarean section remain essential components of safe management in this setting. The existing literature, while limited by the absence of randomized controlled trial data, does not support a universal policy of cesarean delivery for all women after myomectomy when the endometrial cavity was not entered [3,5,8].

## Conclusions

We report a successful vaginal delivery following low-dose oxytocin induction of labor at 40+5 weeks in a 30-year-old primigravida with a prior laparoscopic myomectomy, performed three years earlier, in which the endometrial cavity was preserved and multilayer uterine closure was employed. No uterine rupture, scar dehiscence, neonatal morbidity, or other significant complications were observed. This case adds to the emerging evidence that selected women with post-myomectomy uterine scars may be suitable candidates for the induction of labor with low-dose oxytocin, provided that established risk-stratification criteria are met and continuous intrapartum monitoring is maintained. Further prospective studies are needed to refine patient selection criteria and establish evidence-based protocols for induction of labor in this population.
